# Off-pump or minimized on-pump coronary surgery - initial experience with Circulating Endothelial Cells (CEC) as a supersensitive marker of tissue damage

**DOI:** 10.1186/1749-8090-6-142

**Published:** 2011-10-19

**Authors:** Thorsten Wittwer, Yeong-Hoon Choi, Klaus Neef, Mareike Schink, Anton Sabashnikov, Thorsten Wahlers

**Affiliations:** 1 Department of Cardiothoracic Surgery, Heart Center, University Hospital of Cologne, Germany; 2 Center of Molecular Medicine Cologne, University Hospital of Cologne, Germany

**Keywords:** Minimal Invassive Cardiac Surgery, Minimised Extracorporeal circulation, OPCAB, Circulating endothelial cells

## Abstract

**Background:**

Off-pump-coronary-artery-bypass-grafting (OPCAB) and minimized-extracorporeal-circulation (Mini-HLM) have been proposed to avoid harmful effects of cardiopulmonary-bypass (CPB). Controversies exist whether OPCAB is still superior in perioperative outcome. Circulating endothelial cells (CEC) are sensitive markers of endothelial damage and are significantly elevated in conventional-CPB-procedures as compared to Mini-HLM-revascularisation. Therefore, CEC might be of specific value in evaluating effectiveness of Mini-HLM and OPCAB as currently applied less-invasive coronary procedures.

**Methods:**

76 coronary patients were randomly assigned either to OPCAB (n = 34) or to Mini-HLM (ROCsafe™, Terumo Inc., n = 42) procedures. Perioperative data, clinical and serological outcome and measurements of CEC-release and parameters of endothelial function (v.Willebrand-Factor, soluble-thrombomodulin) perioperatively (pre-operative-baseline, post-Mini-HLM/release of OPCAB-stabilizer, 6 h, 12 h, 24 h and 5 days postoperatively) were obtained and compared by ANOVA models including repeated-measures-analysis.

**Results:**

Mean graft-number was 3.06 ± 0.72 in Mini-HLM-patients and 1.89 ± 0.74 in OPCAB-patients (p < 0.001). However, ventilation-, ICU- and total-hospital duration were comparable between groups as well as chest-tube-drainage, transfusion requirements, hemodynamics and catecholaminergic support (p > 0.05). CEC-release did not differ between groups (p = 0.274) and was generally within normal limits, Troponin-T levels where not significanty different (p = 0.108). No myocardial infarctions, strokes or deaths occurred, neuron specific enolase (NSE) did not show any differences between groups (p = 0.194).

**Conclusion:**

Conceptional advantages of minimized CPB systems (ROCsafe™) result in morbidity and mortality comparable with OPCAB procedures. Mini-HLM therefore minimizes CPB-related systemic and organ injury as demonstrated by low CEC-values which indicates intact endothelial integrity. Furthermore, Mini-HLM combines OPCAB-benefits with low morbidity in high-risk patients while facilitating more complete revascularization in complex patients.

## Introduction

For decades coronary artery bypass grafting (CABG) was performed with the use of conventional cardiopulmonary bypass (CCPB). However, CCPB has been considered to be a potent stimulus of a generalized inflammatory state and thus having the potential to result in significant morbidity [1]. In order to decrease morbidity and mortality associated with coronary surgery, myocardial revascularization without CCPB has been introduced into clinical practice in terms of the off-pump coronary artery bypass grafting (OPCAB) procedure [2]. A number of randomized controlled studies comparing OPCAB to CCPB have been completed since then. Although outcomes have been largely comparable, the evidence of benefit of OPCAB has not been as convincing as primarily anticipated [3]. Technically, OPCAB revascularisation can be very demanding, particularly when marginal branches need to be revascularized which may result in severe hemodynamic instability due to cardiac displacement [4]. Therefore, initial enthusiasm for OPCAB became especially tempered by concern about the completeness of revascularization, the rate of perioperative myocardial infaction and long-term graft patency rates [5,6]. As a consequence, minimized extracorporeal circulation systems (Mini-HLM) have been proposed to avoid the potentially harmful effects of CCPB. The basic idea of Mini-HLM is to ensure adequate perfusion by a closed, extremely minimized circuit based on a rotary blood pump and a high-performance membrane oxygenator with elimination of blood-to-air contact by avoiding a venous reservoir, minimizing hemodilution and mechanical blood trauma and significant reduction of contact activation by reduced foreign surfaces [7]. Meanwhile, a clear superiority of Mini-HLM systems could be proven when compared to conventional CPB circuits [8]. Among the different available minimized systems, the ROCSafe™ systems (Terumo Medical Corp., Somerset, NJ, USA) is associated with superior de-airing, is suitable for both coronary and aortic valve surgery and was shown to improve postoperative recovery, reduce early inflammatory response, transfusion requirements and atrial fibrillation [9,10]. One major mechanism of the beneficial effect of Mini-HLM is considered to be the lesser degree of endothelial injury which can be specifically assessed by quantification of Circulating Endothelial Cells (CEC) which represent a novel marker of the intrinsic endothelial damage caused by cardiopulmonary bypass [11]. Detachment of endothelial cells into the blood stream represents a serious injury of the endothelium as one of multiple severe adverse effects of CCPB [1,11]. As quantification of CEC can unveil both endothelial damage and correlate with activity as well as degree of injury at early preclinical stages [12,13], the combined approach of CEC quantification and cardiac Troponin measurement may significantly improve the diagnostic accuracy in evaluation of different coronary revascularization procedures in analogy to findings in NSTEMI-patients [11,14]. As there are still very few studies available comparing the modern less invasive surgical procedures Mini-HLM- with OPCAB-revascularization [15], it was the aim of our present study to directly compare both currently applied surgical revascularization procedures with special regard to the corresponding kinetics of perioperative CEC release which was not performed in the available literature so far.

## Materials and methods

### 1. Patients

This prospective randomized ethics approved clinical trial was performed between July 2009 and January 2010 at our institution. Included were a total of 76 stable coronary patients (age > 18 years) according to the following criteria: all patients were scheduled for elective isolated myocardial revascularization performed via full median sternotomy and had been judged technically suitable for both OPCAB and Mini-HLM techniques. Indication for coronary surgery was established on the basis of current international guidelines [16]. Patients with unstable angina, myocardial infarction preoperative proinflammatory status, insulin-dependent diabetes or inflammatory vascular diseases were excluded from this study as CEC-values are known to be elevated in all these instances [17]. After inclusion, all patients were randomized according to a computer-generated algorithm either to the OPCAB or the Mini-HLM-procedure. The institutional ethics committee approved this study, and all patients gave informed written consent prior to entering the study.

### 2. Analysis of CEC frequency

**Figure 1:**
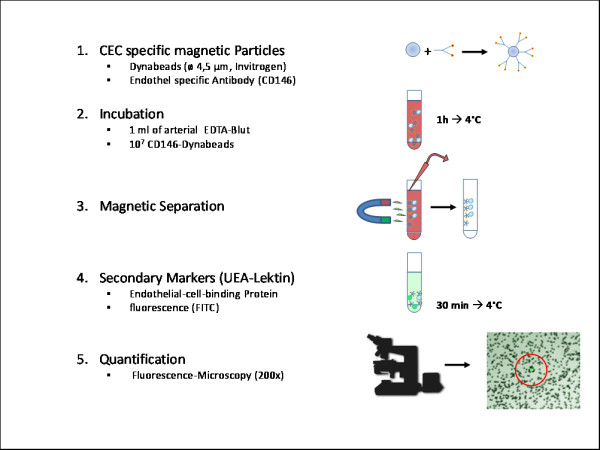
**Separation of circulating endothelial cells**. EDTA blood was incubated with anti-CD146 labelled dynabeads, circulating endothelial cells were magnetically separated and quantified by fluorescence microscopy.

CEC frequency in the peripheral blood was determined as described previously [11] with minor modifications (Figure 1). Briefly, arterial blood samples were collected in 2,7 ml EDTA tubes (Sarstedt, Nümbrecht, Germany), and stored at 4°C for a maximum of 24 h for later batch analysis. The monoclonal mouse anti-human CD146 antibody (clone S-Endo1/F4-35H7, Biocytex, Marseille, France) was conjugated to rat-anti-mouse-IgG1-dynabeads (diameter 4.5 μ m, Invitrogen, Karlsruhe, Germany) according to the manufacturer's instructions.

For Immunomagnetic labeling of CEC the EDTA blood sample was diluted 1:1: with PEB buffer (phosphate buffered saline, PBS, pH 7.4 (Invitrogen), 0.01% bovine serum albumin, BSA (PAA, Cölbe, Germany), 10 mM ethylene-diamine-tetra-acetic acid, EDTA (Carl Roth, Karlsruhe, Germany)) and adding 100 μ l FcR blocker (Miltenyi Biotec, Bergisch-Gladbach, Germany) to prevent unspecific leukocyte binding, and 100 μ l CD146-coupled dynabeads. Samples were incubated on a rotator (10 rpm) for one hour at 4°C. Immunomagnetically labeled cells were isolated in a specific magnetic separator (Dynal MPC-L, Invitrogen). After washing thrice with PEB the isolated cells were resuspended in 90 μ l PEB + 10 μ l fluorescein-labeled Ulex-europaeus-agglutenin-1 (UEA-1, Vector Laboratories, Burlingame, CA, USA) and incubated for 1 h on a shaker (300 rpm) at 4°C in the dark. After three wash cycles in PEB the cells were resuspended in 200 μ l PEB. CEC were identified and enumerated in 50 μ l samples independently by three blinded observers using an inverted fluorescence microscope (Ti-U equipped with a DS-Qi1MC camera, Nikon, Düsseldorf, Germany) at 20x magnification, phase contrast, 10% transmission light and fluorescein excitation.

Criteria defining a CEC [18] were:

1. fluorescein positive

2. 15-30 μ m diameter of cell body and

3. bound to at least 4 dynabeads.

The total number of CEC was normalized to a volume of one ml of peripheral blood

### 3. Serology

**Figure 2:**
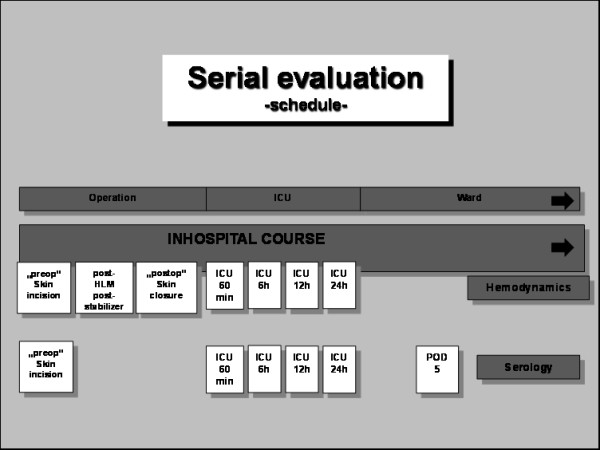
**Schedule of data collection**. Serial evaluation of hemodynamics and serological parameters with regard to hospital stay.

Serological evaluation of patients' blood was performed at six different time points perioperatively (Figure 2) according to standard hospital protocols including cardiac enzymes creatinin kinase (CK), CK-MB, Troponin T and neuron-specific enolase (NSE). Additionally, von-Willebrand factor antigen (vWF) was measured by immunoturbidimetric determination using the Dade Behring vWF:Ag test kit (Dade Behring Marburg GmbH, Marburg, Germany). For determination of soluble thrombomoduline concentration (sTM, CD 141), a commercial solid phase sandwich enzyme-linked immunsorbent assay kit was used (human sCG141 ELISA kit. Diaclone Research, Besancon, France).

### 4. Hemodynamic evaluation

All patients were monitored by invasive hemodynamic assessment using a pulmonary artery catheter. Data collection was performed at 7 different time points perioperatively (Figure 2).

### 5. Statistical Analysis

All data were stored and analyzed using the SPSS statistical package 17.0 (SPSS Inc., Chicago, Ill., USA). Descriptive statistics were computed for variables of interest and analyzed using univariate ANOVA. Continuous data were analyzed using ANOVA with repeated measures. Significance was assumed with a p-value < 0.05.

## Results

**Figure 3:**
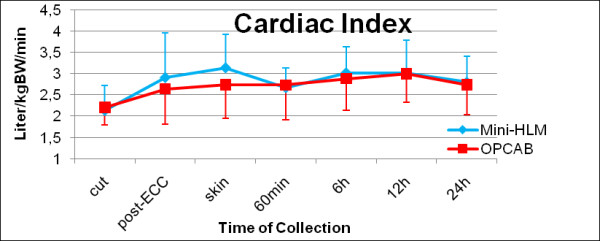
**Invasive hemodynamics: perioperative cardiac index**. Serial time couse of cardiac index measured by pulmonary artery catheter in patients operated by use of Mini-HLM (blue line) or by OPCAB techniques (red line); Significance level: p = 0.504.

Patients' perioperative demographics are summarized in Table 1. There was no statistical difference between both experimental groups regarding age, gender, weight and Euro-Score. Generally, no mortality, perioperative stroke or ST-elevation myocardial infarct was observed during the entire study period. Operation time was significantly longer in the Mini-HLM group (179 ± 34 minutes vs. 141 ± 34 minutes), however perioperative incidence of atrial fibrillation or transitory psychotic disorder syndromes was equally low distributed between groups. Operative usage of bilateral internal mammry artery grafts did not show any significant differences, and overall chest tube drainage, ventilation time, transfusion requirements and total intensive care stay were comparable in both cohorts. Serial assessment of patients' hemodynamics did nor show any differences in cardiac index (p = 0.504, Figure 3).

**Table 1:** Patients' demographics and perioperative data

	Mini-HLM	OPCAB	p-Wert
Age (yrs)	65,6 ± 11,2	64,7 ± 10,9	0,723
Heigh (cm)	173 ± 7	168 ± 8	0,307
Weight (kg)	85,1 ± 12,1	83,7 ± 14,6	0,673
Additive Euroscore	3,1 ± 2,1	3,0 ± 2,0	0,766
**Mortality**	**0**	**0**	
**Stroke**	**0**	**0**	
**STEMI**	**0**	**0**	
Transitory psychotic disorder syndrome	2/42 (4,7%)	1/34 (2,9%)	0,197
Postoperative atrial fibrillation	16/42 (38,1%)	14/34 (41,2%)	0.817
Operation time (minutes)	174,6 ± 33,4	138,9 ± 32,9	< 0.001
LIMA +RIMA usage	11/42 (26,2%)	6/34 (17,6%)	0.419
Intensive care stay (days)	2,55 ± 0,97	2,18 ± 7,3	0,075
Chest tube drainage (48 hours)	1204 ± 600	1040 ± 412	0,187
Ventilation time (hours)	16,3 ± 10,0	13,2 ± 3,9	0,101
Transfusion of Red Blood Cells postoperatively	1,45 ± 1,96	0,81 ± 1,31	0,119
Transfusion of thrombocytes postoperatively	0,35 ± 0,74	0,16 ± 0,45	0,195
Transfusion of Fresh Frozen Plasma postoperatively	0,75 ± 2,1	0,56 ± 1,4	0,65

### Circulating Endothelial Cells

**Figure 4:**
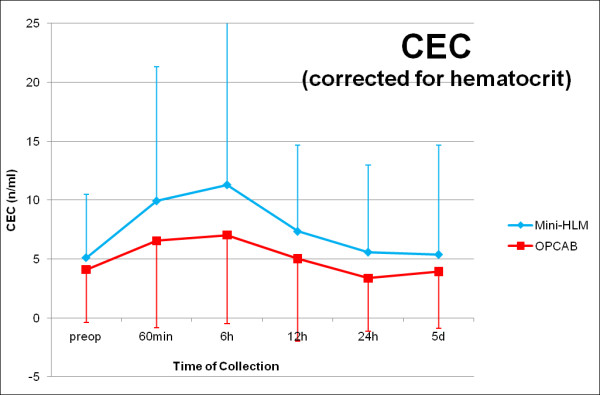
**Perioperative numbers of Circulating Endothelial Cells (CEC, corrected for hematocrit)**. Serial time couse of CEC in patients operated by use of Mini-HLM (blue line) or by OPCAB techniques (red line); Significance level: p = 0.274.

Preoperative CEC numbers (cells per milliliter of blood) did not differ between the experimental groups (Mini-HLM: 7,39 ± 9,94; OPCAB: 7,03 ± 12,54; p = 0.901). 60 minutes after arrival on the ICU, CEC values peaked in both groups and decreased over time until postoperative day 5, where the preoperative niveau was reached (Figure 4). Statistical analysis did not reveal any significant differences regarding the CEC kinetics between both groups (p = 0.274).

### Serology

**Figure 5:**
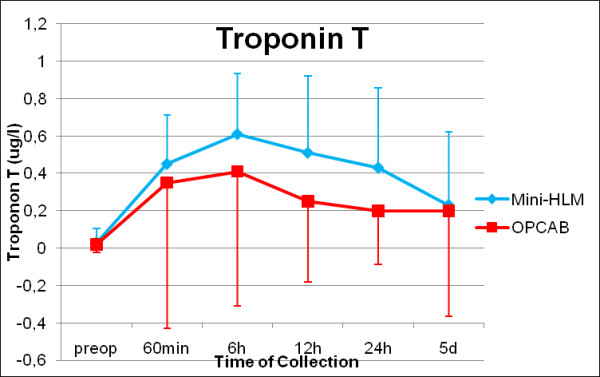
**Cardiax enzymes perioperatively: Troponin T**. Serial time couse of Troponin T in patients operated by use of Mini-HLM (blue line) or by OPCAB techniques (red line); Significance level: p = 0.108.

**Figure 6:**
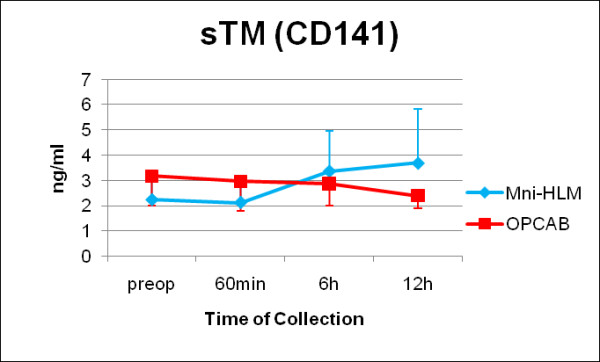
**Serology perioperatively: soluble thrombomodulin (sTM, CD141)**. Serial time couse of sTM in patients operated by use of Mini-HLM (blue line) or by OPCAB techniques (red line); Significance level: p = 0.102.

Serial evaluation of troponin T values (Figure 5, p = 0.108) and NSE did not show any significant differences between Mini-HLM and OPCAB operated patients according to the clinical results of freedom from STEMI and incidence of transitory psychotic disorder syndrome. Furthermore, kinetics of soluble thrombomodulin (p = 0.102, Figure 6) and von Willebrand factor antigen did not show any significant differences.

### Vessel disease and graft number

**Figure 7:**
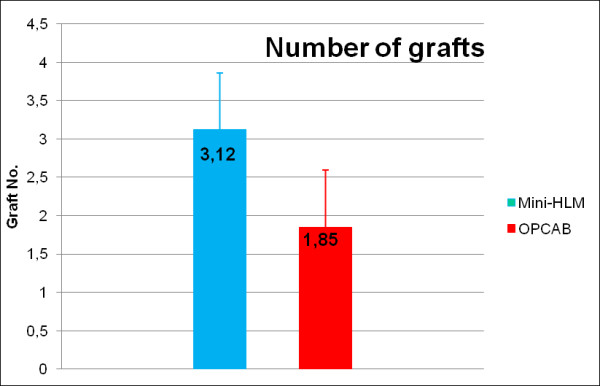
**Coronary artery bypass grafts**. Number of bypass grafts actually performed in Mini-HLM group (blue) or OPCAB group (red). Significance level: p < 0.001.

According to the preoperative angiograms, the degree of vessel disease was equally distributed between groups, and a mean number of 2.72 ± 0.52 grafts was preoperatively planned in Mini-HLM patients in contrast to 2.47 ± 0.84 grafts in OPCAB patients (p = 0.204). Intraoperatively, however, the actual graft number (Figure 7) was significantly higher in the Mini-HLM group (3.06 ± 0.72) as compared to OPCAB operated patients (1.89 ± 0.74, p < 0.01) indicating a more complete revascularization in the Mini-HLM group.

#### Comment

Cardiac surgery performed with CCPB may lead to serious complications in up to 20% of low-risk patients [19]. More than two decades ago, Kirklin et al. [1] reported complement activation following CCPB which triggers a whole body defense reaction which may lead to significant alterations of cerebral function and multiple other harmful effects. After introduction of the minimally invasive direct coronary artery bypass (MIDCAB) procedure for treatment of single vessel (LAD) disease [20], the evolution of that promising off-pump approach approach let to the interdisciplinary multivessel "hybrid procedure" with MIDCAB-grafting of the LAD culprit lesion followed by interventional stenting of remaining coronary lesions [21]. However, long-term outcome of hybrid procedures might be limited by the known restenosis rates of stented areas [22]. The complete surgical multivessel revascularization on the beating heart (OPCAB) was introduced in the mid-1990's [2] and is a safe and well-established technique. Patients who undergo coronary surgery with this strategy were initially shown to have a lower incidence of postoperative complications and remarkable advantages in terms of hospital stay [23]. However, although there was a significantly lower deterioration in psychometric tests in OPCAB patients in the early postoperative course as compared to CCPB patients [24], this advantage of the OPCAB technique has resolved with respect to the 5-year cognitive and cardiac outcomes [25]. Furthermore, complete coronary revascularization may not be achievable in all patients by off-pump techniques owing to the complex anatomy of coronary lesions and the possibility of hemodynamic instability while the beating heart is manipulated [4]. Interestingly, recent studies show inferior long-term patency rates and incompleteness of revascularization with regard to OBCAB-techniques [26,27]. In the recently published ROOBY trial [28], especially the lower patency rate of saphenous vein grafts in the OPCAB group accounted for the observed differences in graft function. However, with special attention to the prognostically important left internal thoracic artery grafts to the LAD culprit lesions, it could be shown that - with classification of those grafts according to the established FitzGibbon grade [29] - there were significantly fewer grade A grafts in the OPCAB group than in the cardiopulmonary bypass group indicating lower quality of graft anastomoses. As a consequence, multiple efforts were taken to achieve the same advantages with modified cardiopulmonary bypass systems as can be achieved with OPCAB approaches. The solution was miniaturization of CBP-systems thus resulting in reduction of foreign surfaces, avoidance of blood-air contact and significant reduction of priming volume. The advantages of such minimized systems have been shown in several clinical studies so far [30,31]. Overall experience indicates an inferior biocompatibility of CCPB compared to Mini-HLM [32] which is considered to be caused by contact activation of blood cells with artificial surfaces and air, the ischemia and reperfusion injury and hemodilution. Furthermore, the endoxemia caused by intestinal hypoperfusion represents a predominant trigger of complement activation and profound endothelial damage [33]. In this context, a modern approach for assessing endothelial integrity includes the determination of circulating endothelial cells (CEC) in the peripheral blood. CEC are defined as mature endothelial cells in the peripheral blood, detached from vessel walls as a result of injury via mechanical strain or disease or inflammation via paracrine or endocrine factors. The correlation of CEC and cardiovascular disease and its implications have recently been reviewed extensively [34]. Under physiologic conditions, CEC occur in humans in the range of 5-10 cells per ml blood, whereas elevated numbers are found in patients with different vascular disorders and type 2 diabetes mellitus [35,36]. The detachment of endothelial cells into the blood stream represents a serious injury of the endothelium as one of multiple severe adverse effects of CCPB [1,11], and overall CEC values are significantly lower in OPCAB patients when compared to standard cardiopulmonary bypass procedures [37]. CEC do not only unveil endothelial damage but also correlate with activity and degree of endothelial injury [12]. Therefore, CEC are considered to represent a novel marker of the intrinsic endothelial damage caused by CCPB, and use of modern Mini-HLM systems were found to be associated with significantly reduced CEC release as compared to CCPB [11].

The main results of this present study indicate that a Mini-HLM approach by means of the ROCSafe™ system can achieve overall clinical results that are completely comparable to those of OPCAB revascularisation. Although non-elective patients and patients with insulin-dependent diabetes mellitus had to be excluded from the study as unstable angina and/or acute myocardial infarction as well as diabetes per se significantly increase CEC numbers [17], no further restrictions were imposed with regard to enrollment, and the study patients therefore represent an institution-based cohort of routine coronary surgical practice. As the development of modern and risk-adjusted concepts for complete and safe revascularization in coronary patients is one of the main goals in coronary surgery, use of Mini-HLM and thus minimizing the side effects of CCPB is a desirable modern approach. In today's economically affected health care systems, this conclusion is especially important as OPCAB procedures are associated with longer hospital stays and greater overall hospitalization costs in significant dimensions [38]. Increasingly, the referring cardiologists or the patients themselves insist on an OPCAB procedure. The medical decision to apply the OPCAB technique in these patients is a delicate balance between handling the pressure to compete for more CABG cases and providing sound surgical care [38]. Therefore, performing OPCAB in every single patient who seems to be a candidate for surgical myocardial revascularisation for the sole purpose of attracting more patients or due to other political and economic pressures may not be appropriate and economically hazardous [38]. With regard to the increasing overall excellent experience with Mini-HLM worldwide [39,40], OPCAB should be restricted to carefully selected special cases, i.e. patients presenting with severely calcified aorta etc..

The described results should be considered provisional and worthy of further investigation in larger studies, because the relatively small sample size might represent a limitation to our conclusions. However, the major finding of the present investigation is the fact that CEC release and, thus, endothelial damage, is completely comparable between Mini-HLM procedures and the OPCAB technique.

## Conclusion

Conceptional advantages of the closed minimized CPB-system ROCsafe™ result in morbidity and mortality comparable with OPCAB procedures. Mini-HLM, therefore, minimizes CPB-related systemic and organ injury as demonstrated by low CEC-values which indicates intact endothelial integrity. Furthermore, Mini-HLM combines OPCAB-benefits with less morbidity in high-risk-patients while facilitating more complete revascularisation in patients with complex lesions. Mini-HLM should therefore be applied as a routine and gold standard technique in coronary artery bypass surgery.

## Abbreviation list

CABG: coronary artery bypass graft; CCPB: conventional cardiopulmonary bypass; CEC: circulating endothelial cells; ICU: intensive care unit; LAD: left anterior descending artery; MIDCAB: minimally invasive direct coronary artery bypass; Mini-HLM: minimized extracorporeal circulation system; NSE: neuron-specific enolase; NSTEMI: non-ST-elevation myocardial infarction; OPCAB: off-pump coronary artery bypass; sTM: soluble thrombomoduline; vWF: von-Willebrand factor.

## Competing interests

The authors declare that they have no competing interests.

## Authors' contributions

TW (first author) created concept and design, performed most surgical procedures, performed data analysis and interpretation, calculated all statistics and drafted the article. YHC participated in the design of the study, performed surgical procedures, revised the manuscript critically and approved the final version. KF participated in the study concept and data analysis, revised the manuscript and approved the final manuscript. MS participated in data analysis, revised the article and approved the final manuscript. AS collected all data, participated in the analysis of data, revised the article and approved the final manuscript. TW (senior author) approved the concept of the study, revised the article and approved the final version. All authors have read and approved the final manuscript.
